# Preoperative biliary drainage in perihilar cholangiocarcinoma: retrospective multicentre analysis

**DOI:** 10.1093/bjsopen/zrag048

**Published:** 2026-06-08

**Authors:** Julien A Luyten, Pim B Olthof, Sander M J van Kuijk, Silvio Nadalin, Hauke Lang, Ruslan Alikhanov, Joris I Erdmann, Johann Pratschke, Shishir K Maithel, Roberto Troisi, Andreas A Schnitzbauer, Ernesto Sparrelid, Peter Lodge, Massimo Malagò, Ulf P Neumann, Erik Schadde, Hassan Z Malik, Keith J Roberts, Baki Topal, Frederik J H Hoogwater, Jeroen Hagendoorn, Andrea Ruzzenente, Concepcion Gomez, William R Jarnagin, Matteo Cescon, Luca Aldrighetti, Bas Groot Koerkamp, Maxime J L Dewulf, Steven W M Olde Damink, Perihilar Cholangiocarcinoma Collaboration Group, J Rolinger, I Capobianco, M Giglio, A Guglielmi, F Bartsch, M Efanov, G Kazemier, B ten Haaft, B Bracke, T Chapelle, C Benzing, M Schmelzle, S Buettner, R J Porte, W O Bechstein, J Heil, S Gilg, H Jansson, S A W Bouwense, P de Reuver, E de Savornin Lohman, S van Laarhoven, J Bednarsch, L M Quinn, R Sutcliffe, D M Bartlett, J Jaekers, H Topal, M T de Boer, I Q Molenaar, E Poletto, M Ravaioli, M Serenari, C Dopazo, M I D'Angelica, T P Kingham, R Marino, F Ratti

**Affiliations:** Department of Surgery, Maastricht University Medical Centre, Maastricht, the Netherlands; NUTRIM, School of Nutrition and Translational Research in Metabolism, Maastricht University, Maastricht, the Netherlands; Department of Hepatobiliary, Endocrine and Transplantation Surgery, Antwerp University Hospital, Edegem, Belgium; Department of Surgery, Erasmus MC Cancer Institute, Rotterdam, the Netherlands; Department of Surgery, Amsterdam UMC, Amsterdam, the Netherlands; Department of Clinical Epidemiology and Evaluation of Medical Technology, Maastricht University Medical Centre, Maastricht, the Netherlands; Department of General and Transplant Surgery, University Hospital Tübingen, Tübingen, Germany; Department of General, Visceral and Transplantation Surgery, University Medical Center Mainz, Mainz, Germany; Department of Hepato-Pancreato-Biliary Surgery, Moscow Clinical Scientific Center, Moscow, Russia; Department of Surgery, Cancer Center Amsterdam, Amsterdam UMC, University of Amsterdam, Amsterdam, the Netherlands; Department of Surgery, Campus Charité Mitte, Campus Virchow-Klinikum, Experimental Surgery and Regenerative Medicine, Charité, Berlin, Germany; Division of Surgical Oncology, Department of Surgery, Winship Cancer Institute, Emory University, Atlanta, Georgia, USA; Division of Hepato-Bilio-Pancreatic, Minimally Invasive and Robotic Surgery, Department of Clinical Medicine and Surgery, Federico II University Hospital, Naples, Italy; Department of General and Visceral Surgery, University Hospital, Goethe University, Frankfurt, Germany; Division of Surgery and Oncology, Department of Clinical Science, Intervention and Technology, Karolinska Institutet, Karolinska University Hospital, Stockholm, Sweden; HPB and Transplant Unit, St James’s University Hospital, Leeds Teaching Hospitals NHS Trust, Leeds, UK; Department of Hepatopancreatobiliary and Liver Transplantation Surgery, University College London, Royal Free Hospitals, London, UK; Department of General, Visceral, Vascular and Transplantation Surgery, University Hospital Essen, Essen, Germany; Department of Surgery, Cantonal Hospital Winterthur, Zurich, Switzerland; Department of Hepatobiliary Surgery, Aintree University Hospital, Liverpool University Hospitals, NHS Foundation Trust, Liverpool, UK; Department of Surgery, University Hospital Birmingham, Birmingham, UK; Abdominal Surgery, UZ Leuven, Leuven, Belgium; Department of Hepato-Pancreato-Biliary Surgery and Liver Transplantation, University Medical Center Groningen, Groningen, the Netherlands; Department of Surgery, Regional Academic Cancer Centre Utrecht, St Antonius Hospital, Nieuwegein and University Medical Centre Utrecht, Utrecht, the Netherlands; Department of Surgery, Unit of Hepato-Pancreato-Biliary Surgery, University of Verona Medical School, Verona, Italy; Department of Hepatopancreatobiliary Surgery and Transplants, Vall D’Hebron Hospital Universitari, Vall D’Hebron Institut de Recerca (VHIR), Vall D’Hebron Barcelona Hospital Campus, Universitat Autónoma de Barcelona, Barcelona, Spain; Hepatopancreatobiliary Service, Department of Surgery, Memorial Sloan Kettering Cancer Center, New York, New York, USA; General Surgery and Transplantation Unit, Azienda Ospedaliero-Universitaria di Bologna, Bologna, Italy; Hepato-Biliary Surgery Division, Ospedale San Raffaele-IRCCS, Milan, Italy; Department of Surgery, Erasmus MC Cancer Institute, Rotterdam, the Netherlands; Department of Surgery, Maastricht University Medical Centre, Maastricht, the Netherlands; Department of Surgery, Maastricht University Medical Centre, Maastricht, the Netherlands; NUTRIM, School of Nutrition and Translational Research in Metabolism, Maastricht University, Maastricht, the Netherlands; Department of General, Visceral, Vascular and Transplantation Surgery, University Hospital Essen, Essen, Germany

## Abstract

**Background:**

There is no consensus on the use of preoperative biliary drainage for resectable perihilar cholangiocarcinoma. This retrospective cohort study aimed to explore the association of biliary drainage with postoperative mortality and morbidity.

**Methods:**

This retrospective observational cohort study included patients who underwent resection of histologically confirmed perihilar cholangiocarcinoma from the Perihilar Cholangiocarcinoma Collaboration Group database across 27 Western hepatobiliary centres (2000–2022). To correct for baseline differences between patients who did or did not undergo drainage, propensity score weighting was applied. Outcomes were compared using propensity score-weighted regression and multivariable analysis.

**Results:**

Overall, 2067 patients were included, of whom 350 (16.9%) did not undergo biliary drainage. Before propensity score weighting, patients who did not undergo drainage were less likely to have Bismuth III–IV disease (297 (78.9%) *versus* 1448 (84.3%); *P* < 0.001), had lower median bilirubin levels (12.0 *versus* 85.5 µmol/l; *P* < 0.001), and a higher proportion had left hepatectomies (150 (42.9%) *versus* 454 (26.4%); *P* < 0.001). After propensity score-weighted regression analysis, patients in the drainage group were more likely to experience major postoperative complications (odds ratio 1.43, 95% confidence interval 1.04 to 1.95; *P* = 0.027) and posthepatectomy liver failure (odds ratio 2.12, 1.25 to 3.58; *P* = 0.005). In multivariable analysis, only posthepatectomy liver failure remained significant (odds ratio 2.13, 1.29 to 3.54; *P* = 0.003).

**Conclusion:**

In this retrospective propensity score weighting analysis, preoperative biliary drainage was associated with a higher incidence of posthepatectomy liver failure in resectable perihilar cholangiocarcinoma. These findings suggest that a subgroup of patients with perihilar cholangiocarcinoma can be operated safely without biliary drainage. The indication for preoperative biliary drainage should be considered on an individual basis.

## Teaser

This retrospective multicentre cohort study evaluated the impact of preoperative biliary drainage on postoperative outcomes in patients undergoing resection with curative intent for perihilar cholangiocarcinoma. Leveraging data from 27 international high-volume hepatobiliary centres, and using both propensity score weighting and multivariable regression, it was demonstrated that preoperative biliary drainage is associated with a significantly higher risk of posthepatectomy liver failure. These findings question the routine use of biliary drainage and support further prospective evaluation of drainage-free surgical strategies in selected patients.

## Introduction

Perihilar cholangiocarcinoma (pCCA) represents the predominant subtype of biliary tract cancer, emerging at the junction of the hepatic ducts^1,2^. Radical surgical resection achieves a median overall survival (OS) of 30–45 months^2,3^. The Nagoya group^4^ has demonstrated a postoperative mortality rate below 2% and Western centres report 90-day mortality rates of 8–18%, with more than half of patients experiencing major postoperative complications^5^. Despite these risks, aggressive surgical management is justified, as palliative systemic therapy offers a median OS of 13 months^6^.

Given these risks, optimal preoperative management is essential^2^. Preoperative biliary drainage (BD) is commonly performed in patients with obstructive jaundice and is considered mandatory before liver hypertrophy-inducing procedures^1,7^. BD can be achieved via endoscopic retrograde cholangiopancreatography (ERCP) or percutaneous transhepatic BD (PTBD)^8^.

Drainage-induced cholangitis is the most severe complication in pCCA, and frequently necessitates antibiotic treatment and repeated interventions^9,10^. Preoperative cholangitis is associated with prolonged hospital stay, deterioration of performance status, and reduced resectability, as illustrated by nationwide data showing low surgical rates among patients with pCCA^2^. Before surgery, BD is also associated with a high mortality rate ranging from 11 to 46%^10,11^; cholangitis is the most common complication and a likely contributor to these outcomes. Preoperative cholangitis can also increase the risk of morbidity and mortality after surgery, predominantly owing to posthepatectomy liver failure (PHLF)^5,12,13^.

Several retrospective studies^14–16^ have failed to demonstrate a clear improvement in postoperative outcomes with routine BD compared with no drainage, except in selected subgroups^17^. This retrospective cohort study aimed to explore the association between preoperative BD and postoperative outcomes in patients with pCCA.

## Methods

### Study design

Data from the pCCA Collaboration Group were analysed in this retrospective cohort study. Data on patients with histologically confirmed pCCA, who underwent surgical resection in 1 of 27 hepatobiliary centres between 2000 and 2022, were used. All participating European or North American centres are expert centres, with a median yearly volume of six resections during the inclusion period. The data set included information on patient characteristics, disease presentation, biochemical parameters, preoperative management, surgery, and postoperative outcomes. Tumour node metastasis (TNM) staging was done according to the seventh edition of the Union for International Cancer Control, as the inclusion period preceded the release of the eighth edition.

Ethical approval was not required for this study, as it involved only retrospective analysis of anonymized data. Approval for data use was obtained from the pCCA Collaboration Group.

### Patients

All patients who underwent hepatectomy with histologically confirmed pCCA before surgery or on histopathological examination of the resected specimen were considered eligible for inclusion. Patients were excluded if they underwent isolated bile duct resection or liver transplantation, or if they had missing data for the primary endpoint or preoperative BD status. Patients were grouped according to whether they underwent preoperative BD.

### Outcomes

The primary endpoint was 90-day postoperative mortality, defined as any death occurring within 90 days following surgery. Secondary outcomes included major postoperative complications, classified as ≥ grade III according to the Clavien–Dindo scale^18^, PHLF, postoperative bile leak, and postoperative bleeding, each defined by International Study Group of Liver Surgery criteria^19–21^. Grade B and C were considered clinically relevant for the latter three outcomes. Duration of operation and estimated intraoperative blood loss were considered.

### Analysis

To ensure equivalence between the two groups, propensity score weighting (PSW) was employed. PSW requires a complete data set and, because some weighting and secondary outcome variables had missing data, imputation was done with the mice package in R (R Foundation for Statistical Computing, Vienna, Austria)^22^. Propensity scores were calculated using a generalized linear model. The decision to use weighting instead of matching was made to retain the full data set, as matching would have necessitated the exclusion of considerable numbers of patients, potentially introducing bias. It is important to recognize that PSW produces a weighted pseudopopulation rather than a cohort of individual patients, which adds complexity to interpretation of the findings. Variables for the propensity score model were selected using a two-step approach: initial identification based on baseline differences, followed by selection of clinically relevant confounders suitable for PSW. The twang package^23^ in R was used for calculation.

### Statistical analysis

Categorical variables are presented as absolute numbers with percentages, and continuous variables as mean or median (range), depending on normality assessment based on the Shapiro–Wilk test. Differences between the two groups were tested using Pearson’s χ^2^ test and the Mann–Whitney *U* test, as appropriate. *P* ≤ 0.050 was considered statistically significant. Statistical analyses were undertaken using SPSS^®^ version 28 (IBM, Armonk, NY, USA), R version 3.5.3, and RStudio^®^ version 1.1.463 (RStudio, Boston, MA, USA). Tables and figures were generated using Microsoft^®^ Excel^®^ (Microsoft, Redmond, WA, USA) and Miro (Miro, San Francisco, CA, USA) respectively.

## Results

### Patients and baseline characteristics

**Fig. 1:**
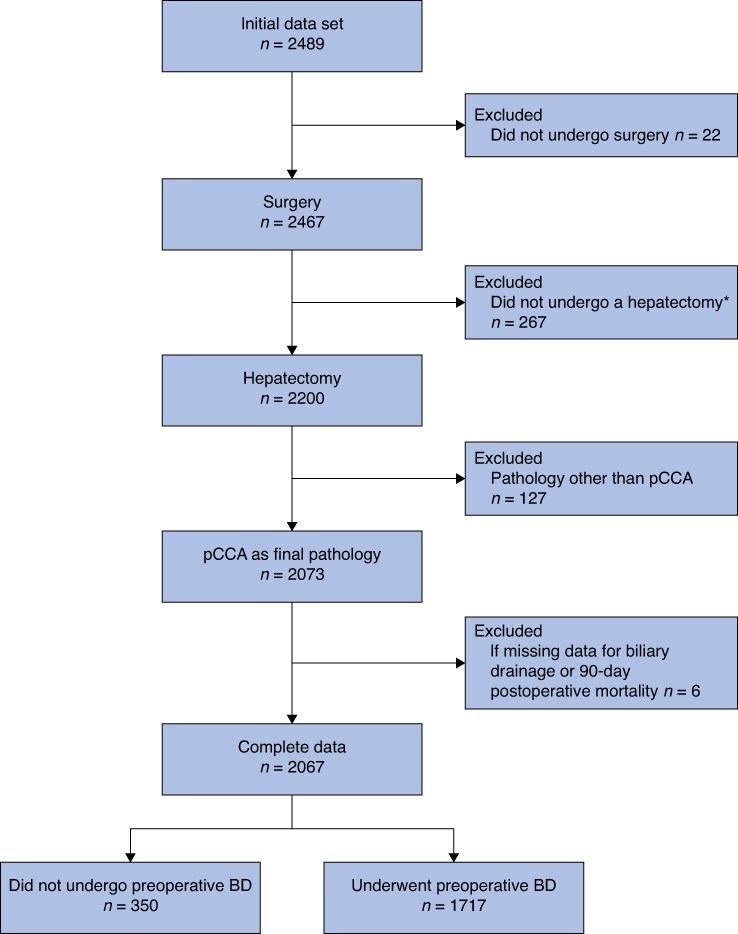
Flow chart showing patient selection from the pCCA Collaboration Group database *Patients undergoing isolated bile duct resection or liver transplant. pCCA, perihilar cholangiocarcinoma; BD, biliary drainage.

Following patient selection (*Fig. 1*), the final data set included information from 2067 patients, of whom 350 (16.9%) did not undergo preoperative BD. Among the group that had drainage, 490 patients (28.6%) underwent percutaneous PTBD, 933 (54.3%) had ERCP, and 294 (17.1% ) underwent both. *Table 1* shows baseline characteristics for both groups.

**Table 1:** Baseline characteristics before and after PSW

	Before PSW			After PSW		
	No drainage (*n* = 350)	Drainage (*n* = 1717)	*P*	No drainage (*n* = 2051.18)*	Drainage (*n* = 2067.83)*	*P*
Age (years), median (i.q.r.)†	68 (58–75)	65 (57–72)	<0.001§	64 (55–73)	65 (57–72)	0.623§
**Sex**†						0.836
Female	144 (41.1%)	714 (41.6)	0.926	(40.7%)	(41.5%)	
Male	206 (58.9%)	1003 (58.4%)		(59.3%)	(58.5%)	
BMI (kg/m^2^), median (i.q.r.)	26.0 (23.4–29.1)	25.0 (22.6–28.2)	<0.001§	26.0 (23.0–29.07)	25.0 (22.8–28.3)	0.149§
ASA grade III + IV†	163 (46.6%)	626 (36.5%)	<0.001	(43.0%)	(38.4%)	0.213
Bilirubin at presentation > 150 µmol/l or 8.8 mg/dl†	23 (7%)	403 (23.5%)	<0.001	(21.9%)	(20.6%)	0.752
Bilirubin at presentation (µmol/l), median (i.q.r.)	12.0 (7.0–37.6)	85.5 (19.0–198.9)	<0.001§	17.0 (8.6–153.9)	73.0 (18.0–75.0)	<0.001§
Bilirubin at presentation (mg/dl), median (i.q.r.)	0.7 (0.41–2.2)	5.0 (1.11–11.6)		1.0 (0.5–9)	4.3 (1.1–4.4)	
Preoperative bilirubin (µmol/l), median (i.q.r.)	12.0 (7.0–38.5)	21.0 (9.0–43.0)	<0.001§	15.0 (8.0–63.0)	20.5 (8.6–41.6)	0.855§
Preoperative bilirubin (mg/dl), median (i.q.r.)	0.7 (0.41–2.25)	1.23 (0.53–2.51)		0.88 (0.47–3.68)	1.20 (0.5–2.43)	
Primary sclerosing cholangitis	9 (3%)	46 (3%)	0.643	(4%)	(3%)	0.795
CA19-9 at presentation or preoperative value (units/ml), median (i.q.r.)	126 (37–377)	149 (41–516)	0.976§	120 (30–397)	145 (40–497)	0.428§
Preoperative cholangitis	12 (3%)	432 (25.2%)	<0.001	(4%)	(24.6%)	<0.001
Bismuth–Corlette classification III + IV†	297 (78.9%)	1448 (84.3%)	0.015	(84.7%)	(83.4%)	0.591
Tumour category T3 + T4 (7th edition)	127 (36.2%)	614 (35.8%)	<0.001	(38.8%)	(35.4%)	0.343
Node category N1 + N2 (7th edition)	146 (41.7%)	745 (40.4%)	0.496	(45.5%)	(42.7%)	0.604
Metastasis category M1(7th edition)	11 (3%)	76 (4%)	0.303	(5%)	(5%)	0.992
Differentiation grade moderate–well	237 (67.7%)	1173 (68.3%)	0.070	(29.6%)	(31.5%)	0.547
Perineural invasion	217 (62.0%)	240 (14.0%)	0.015	(79.5%)	(77.5%)	0.488
**Preoperative biliary drainage**			<0.001			<0.001
None	350 (100%)	0 (0)		(100%)	(0%)	
PTBD	0 (0)	490 (28.6%)		0 (0)	(28.9%)	
ERCP	0 (0)	933 (54.3%)		0 (0)	(54.8%)	
Both	0 (0)	294 (17.1%)		0 (0)	(16.3%)	
Portal vein embolization†	30 (9%)	380 (22.1%)	<0.001	(17.1%)	(19.8%)	0.480
**Type of hepatectomy**†			<0.001			0.993
Left hemihepatectomy	150 (42.9%)	454 (26.4%)		(29.8%)	(29.2%)	
Left extended hemihepatectomy	57 (17%)	291 (17.0%)		(17.1%)	(16.8%)	
Right hemihepatectomy	64 (18%)	294 (17.1%)		(17.9%)	(17.3%)	
Right extended hemihepatectomy	66 (19%)	618 (36.0%)		(32.3%)	(33.1%)	
Segment IV/V	12 (3%)	42 (3%)		(2%)	(3%)	
Central or mesohepatectomy	1 (<1%)	18 (1%)		(0.6%)	(1%)	
Segment I resected	238 (68.0%)	1022 (59.5%)	0.140	(72.8%)	(75.1%)	0.529
Pancreatoduodenectomy	5 (1%)	29 (2%)	0.892	(2%)	(1.75%)	0.918
Portal vein reconstruction†	75 (21%)	604 (40.4%)	<0.001	(35.9%)	(37.2%)	0.728
Hepatic artery reconstruction†	11 (3%)	71 (4%)	0.075	(6.2%)	(4%)	0.207

Values are *n* (%) unless otherwise stated. *Value in the weighted population after propensity score weighting (PSW) reflects the sum of patient weights rather than the actual number of individual patients. †Variables used for PSW. i.q.r., Interquartile range; BMI, body mass index; ASA, American Society of Anesthesiologists; CA, carbohydrate antigen; PTBD, percutaneous transhepatic biliary drainage; ERCP, endoscopic retrograde cholangiopancreatography. ‡Pearson’s χ^2^ test, except §Mann–Whitney *U* test.

Prior to applying PSW, undrained patients less frequently presented with bilirubin levels exceeding 150 µmol/L (8.8 mg/dL, 23 vs 403, *P* < 0.001) and had a lower prevalence of preoperative cholangitis (12 vs. 432, *P* < 0.001). The undrained group had less advanced disease stages (Bismuth III-IV 297 vs. 1448, *P* = 0.015) and underwent PVE less often (30 vs. 380, *P* < 0.001). Undrained patients more frequently underwent left hemihepatectomies (150 vs. 454, *P* < 0.001). Portal reconstruction was less frequent in undrained patients (75 vs. 694, *P* < 0.001).

Following PSW, the proportion of undrained patients with bilirubin levels exceeding 150 µmol/L (8.8 mg/dL) at initial presentation was similar between the groups (21.9% vs. 20.6%, *P* = 0.75). When assessed as a continuous variable, bilirubin at presentation showed an SMD of 0.18 after weighting (*Fig. S1*). Advanced disease stage was also equivalent (Bismuth III-IV: 84.7% vs. 83.4%, *P* = 0.59), as well as the proportions of left hepatectomies and right extended hemi-hepatectomies (29.8% vs. 29.2% and 32.3% vs. 33.1%, resp.; *P* = 0.99). No differences were noted in percentages of PVE procedure (*P* = 0.48) or PV reconstruction (*P* = 0.73). Preoperative cholangitis was not used to determine the propensity score weights, and its incidence remained lower in the undrained group after PSW (4.42% vs 24.64%, *P* < 0.001).

### Postoperative outcomes

Postoperative morbidity and mortality are shown in *Table 2*. Before PSW, the 90-day postoperative mortality rate was lower in the undrained group compared to the drained group (33 vs. 240, *P* = 0.027), as well as major postoperative complications (Clavien-Dindo ≥ III, 126 vs. 880, *P* < 0.001). The undrained group also had lower incidences of PHLF (26 vs. 285, *P* = 0.002) and postoperative bleeding (22 vs. 195, *P* = 0.006). no differences were observed in postoperative biliary leaks (*P* = 0.084).

**Table 2:** Postoperative outcomes before and after PSW

	Before PSW			After PSW		
	No drainage (*n* = 350)	Drainage (*n* = 1717)	*P*†	No drainage (*n* = 2051.18)*	Drainage (*n* = 2067.83)*	*P*†
90-day postoperative mortality	33 (9%)	240 (14.0%)	0.027	(9.8%)	(13.7%)	0.110
Major postoperative complication (Clavien–Dindo ≥ III)	126 (36.0%)	880 (51.3%)	<0.001	(41.5%)	(50.3%)	0.027
PHLF grade B + C#	26 (7%)	285 (16.6%)	0.002	(8.2%)	(15.9%)	0.004
Postoperative bile leak grade B + C#	57 (16.3%)	352 (20.5%)	0.084	(15.9%)	(20.3%)	0.208
Postoperative bleeding grade B + C#	22 (6%)	195 (11.4%)	0.006	(7.4%)	(10.9%)	0.150
Operative blood loss (ml), median (i.q.r.)	700 (400–1175)	840 (500–1600)	<0.001‡	700 (500–1400)	800 (500–1530)	0.094‡
Duration of operation (min), median (i.q.r.)	420 (311.5–5010)	430 (353–525)	0.003‡	420 (324–505	428 (352–525)	0.042‡

Values are *n* (%) unless otherwise stated. *Value in the weighted population after propensity score weighting (PSW) reflects the sum of patient weights rather than the actual number of individual patients. #As defined by International Study Group of Liver Surgery. PSW, propensity score weighting; PHLF, posthepatectomy liver failure; i.q.r., interquartile range. †Pearson’s χ^2^ test, except ‡Mann–Whitney *U* test.

After PSW, the difference in 90-day mortality rate between the groups became non-significant (9.8% vs. 13.7%, *P* = 0.11). the undrained group continued to show a lower incidence of major postoperative complications (41.5% vs 50.3%, *P* =0.027) and of PHLF (7.7% vs 15.5%, *P* = 0.004). There was no difference in postoperative biliary leaks (*P* =0.21) or bleeding (*P* =0.15).

### Propensity score-weighted regression (PSWR) and multivariable analysis

The results of the PSWR analysis are shown in *Table 3*. Patients who underwent BD did not have a significantly higher chance of death within 90 days of surgery (odds ratio (OR) 1.46, 95% confidence interval 0.92 to 2.34; *P* = 0.112), but they had a higher likelihood of major complications (Clavien–Dindo ≥ III: OR 1.43, 1.04 to 1.95; *P* = 0.027). This group also had a higher likelihood of clinically relevant PHLF (OR 2.12, 1.25 to 3.58; *P* = 0.005). Although patients who underwent drainage had higher odds of clinically relevant bile leaks (OR 1.35, 0.85 to 2.15; *P* = 0.209) and postoperative bleeding (OR 1.54, 0.85 to 2.77; *P* = 0.152), these differences were not statistically significant.

**Table 3:** PSWR and multivariable analysis comparing effect of drainage *versus* no drainage on primary and secondary outcomes

	Adjusted odds ratio¶	*P*
PSWR*		
90-day postoperative mortality	1.46 (0.92, 2.34)	0.112
Major postoperative complication (Clavien–Dindo ≥ III)‡	1.43 (1.04, 1.95)	0.027
PHLF grade B + C‡§	2.12 (1.25, 3.58)	0.005
Postoperative bile leak grade B + C‡§	1.35 (0.85, 2.15)	0.209
Postoperative bleeding grade B + C‡§	1.54 (0.85, 2.77)	0.152
**Multivariable analysis**†		
PHLF grade B + C‡§	2.13 (1.29, 3.54)	0.003
Major postoperative complication (Clavien–Dindo ≥ III)‡	1.21 (0.85, 1.73)	0.291

Values in parentheses are 95% confidence intervals. *Survey-weighted logistic models; †for significant outcomes after propensity score-weighted regression (PSWR). ‡Imputed variables. §As defined by the International Study Group of Liver Surgery. ¶Analysis adjusted for age (> 65 years), sex (female), body mass index (> 30 kg/m^2^), American Society of Anesthesiologists grade (I–II *versus* III–IV), primary sclerosing cholangitis, bilirubin at presentation (threshold 150 µmol/l or 8.8 mg/dl), preoperative cholangitis, carbohydrate antigen 19-9 levels (> 100 units/ml), type of hepatectomy (major *versus* minor), resection of segment I, pancreatoduodenectomy, portal venous embolization, portal venous reconstruction, hepatic artery reconstruction, Bismuth–Corlette classification (I–II *versus* III–IV), and preoperative biliary drainage. For full univariable and multivariable analyses please refer to *Table S1*. PHLF, posthepatectomy liver failure.

Because major postoperative complications and clinically relevant PHLF remained more prevalent after PSWR in the group that underwent drainage, univariable and multivariable analyses were conducted (*Table S1*). For major postoperative complications, the isolated effect of preoperative BD was non-significant (OR 1.21, 0.85 to 1.73; *P* = 0.291). For clinically relevant PHLF, the isolated effect of preoperative BD remained significant, with an OR similar to that observed in the PSWR (OR 2.13, 1.29 to 3.54; *P* = 0.003).

## Discussion

Randomized clinical trials comparing upfront surgical resection with and without preoperative BD in patients with pCCA have not been conducted. Most research on this topic is retrospective and relies on techniques such as PSW to ensure comparability between groups^24^. In the present study, before applying PSW, there were large differences in baseline characteristics and postoperative outcomes between groups of patients with pCCA who did or did not undergo BD (disease stage, bilirubin levels, preoperative cholangitis, PVE, portal vein reconstruction). Higher rates of preoperative cholangitis in the drained group remained after PSW, as it was intentionally excluded from the PSW model. This decision was made because cholangitis is usually a consequence of BD rather than an indication for it, and the data set did not distinguish between cholangitis at presentation and that developing after drainage. As such, it can act as a mediator and including it in the model would not have been appropriate.

In terms of outcomes, before PSW, postoperative morbidity and mortality rates were higher in the drained group. Ninety-day postoperative mortality, major postoperative complications, PHLF, and bleeding were more common among patients who underwent BD. Expect for PHLF, they became non-significant after PSW(R) and multivariable analysis, suggesting that the variations in outcomes were due to baseline differences rather than related to BD. PHLF rates remained significantly lower in the undrained group, highlighting the potential link between BD and an increased risk of PHLF.

However, an alternative interpretation should be considered. The group without drainage had markedly lower bilirubin levels at baseline, reflecting less severe cholestasis. The lower PHLF rates may therefore also relate to this more favourable physiological status rather than to the absence of drainage itself. This is consistent with expert practice, whereby surgery is undertaken only once the bilirubin level is below 35 µmol/l (2 mg/dl)^4^. In addition, multivariable analysis of a PSW cohort may also suffer from residual confounding.

The baseline characteristics and outcomes of this full cohort align with those described in a benchmark study^25^, suggesting that the present findings are clinically applicable. These results showed some variation from those reported by Farges *et al*.^17^ in terms of Bismuth–Corlette classification and portal vein reconstruction, whereas both studies identified differences in cholangitis, jaundice, bilirubin levels, and hepatectomy type. These additional findings may be attributed to the larger sample size in the present study, allowing more detailed detection of differences.

A recent study by Sarkhampee *et al*.^24^ showed similar baseline differences between drained and undrained patients with pCCA, but they reported substantially higher bilirubin levels at presentation and before surgery for both drained and undrained groups compared with values in the present study. That study reported higher rates of postoperative morbidity and mortality in the undrained group. It could be argued that, despite matching, the difference in bilirubin levels before surgery, which remained raised in both groups, most notably in patients without drainage, may have had a negative impact on outcomes among those who did not undergo BD.

The primary rationale for preoperative BD is to restore bile flow, and consequentially reduce hepatic and serum accumulation of cholephiles such as bilirubin, which serve as a marker of biliary obstruction and cholestasis. Several experimental studies^26,27^ have reported improved liver regeneration when the enterohepatic circulation is restored before hepatectomy. Clinically, a raised bilirubin level has been linked to a higher risk of postoperative complications, including PHLF^28–31^. Drainage-related complications, such as cholangitis, also increase postoperative morbidity and mortality among patients with pCCA^9^. Cholangitis can also weaken patients, both before and after surgery, and prolong the time from diagnosis to surgery compared with upfront resection^10,12,13,32^.

Several limitations of this study should be acknowledged. The multicentre design means that all treatment decisions were made according to local protocols and expertise, inherently introducing variation in the indication, bilirubin cut-off value, and technique for BD, use of liver hypertrophy-inducing procedures, perioperative antibiotic regimens, and operative strategies. The present data set included only patients who underwent surgery for pCCA and does not account for those who became inoperable owing to complications from BD. Although the data set was comprehensive, certain confounders may not have been accounted for, as it does not include all potentially relevant variables. Insufficient data on future liver remnant volume prevented any adjustments to account for its effect on PHLF. Missing data were addressed through imputation, which, although not without potential bias, is preferable to limiting the analysis to patients with complete data. The latter results in less statistical power and a higher likelihood of biased results. Although most variables had only single-digit percentages of missing data, bilirubin level at presentation had approximately 30% missing data. As such, the impact of imputation was more substantial, which in turn may have affected the robustness and generalizability of the weighting and introduced residual confounding. Although PSW is an artificial method for achieving equivalence, data interpretation should be approached with caution because of severe baseline differences between the two groups. The different sample size of undrained cohort after and before PSW, (350 *versus* 2000) indicated a substantial weighting to achieve balance, and it underscored the artificial nature of the comparability and limits the external validity of the results. As no universal bilirubin threshold exists, any cut-off applied in PSW is inherently arbitrary. Dichotomizing the bilirubin level led to residual confounding as shown in the Love plot (*Fig. S1*). As such, bilirubin level at presentation showed no difference between groups after PSW when analysed as a dichotomized variable, but it remained significantly different when assessed as a continuous variable.

This analysis has not demonstrated that direct surgery is equivalent to preoperative BD followed by surgery, yet it has indicated that some patients may undergo upfront resection without clear evidence of worse outcomes despite the different biases. Other potential advantages of upfront surgical resection, although speculative, include a reduced need for preoperative procedures and a shorter time to surgery. At present, real-world data do not support these assumptions. Preventing non-operability owing to drainage-related cholangitis may also be a consideration, but this cannot be confirmed from the present analysis.

Interpretation should be cautious because of the use of PSW and inherent differences between the groups with and without drainage; nevertheless, surgery without preoperative BD may be feasible in carefully selected patients, such as those without cholangitis, with bilirubin levels below a defined threshold (for example < 200 µmol/l), preserved performance status, an adequate future liver remnant (for example > 40%), and adequate renal function. Although the present findings do not argue against preoperative BD in patients with clear indications, the potential additional risk associated with routine drainage supports a more selective approach and underscores the need for prospective studies to guide clinical decision-making.

## Supplementary Material

zrag048_Supplementary_Data

## Data Availability

The data underlying this article were provided by the pCCA Collaboration Group permission. Data will be shared on request to the corresponding author with permission of the pCCA Collaboration Group.

## Collaborators

J. Rolinger (University Hospital Tübingen, Tübingen, Germany); I. Capobianco, M. Giglio, A. Guglielmi (University of Verona Medical School, Verona, Italy); F. Bartsch (University Medical Center Mainz, Mainz, Germany); M. Efanov (Moscow Clinical Scientific Center, Moscow, Russia); G. Kazemier, B. ten Haaft (Amsterdam UMC, Amsterdam, the Netherlands); B. Bracke, T. Chapelle (Antwerp University Hospital, Edegem, Belgium); C. Benzing, M. Schmelzle (Charité, Berlin, Germany); S. Buettner, R.J. Porte (Erasmus MC Cancer Institute, Rotterdam, the Netherlands); W.O. Bechstein, J. Heil (University Hospital, Goethe University, Frankfurt, Germany); S. Gilg, H. Jansson (Karolinska University Hospital, Stockholm, Sweden); S.A.W. Bouwense (Maastricht University Medical Centre, Maastricht, the Netherlands); P. de Reuver, E. de Savornin Lohman (Radboud University Medical Center, Nijmegen, the Netherlands); S. van Laarhoven (University College London, Royal Free Hospitals, London, UK); J. Bednarsch (University Hospital Essen, Essen, Germany); L.M. Quinn (Liverpool University Hospitals, Liverpool, UK); R. Sutcliffe, D.M. Bartlett (University Hospital Birmingham, Birmingham, UK); J. Jaekers, H. Topal (UZ Leuven, Leuven, Belgium); M.T. de Boer (University Medical Center Groningen, Groningen, the Netherlands); I.Q. Molenaar (University Medical Centre Utrecht, Utrecht, the Netherlands); E. Poletto, M. Ravaioli, M. Serenari (Azienda Ospedaliero-Universitaria di Bologna, Bologna, Italy); C. Dopazo (Vall d'Hebron Hospital Universitari, Barcelona, Spain); M.I. D'Angelica, T.P. Kingham (Memorial Sloan Kettering Cancer Center, New York, USA); R. Marino, F. Ratti (Ospedale San Raffaele-IRCCS, Milan, Italy).
