# Supplementary material for: Preoperative biliary drainage in perihilar cholangiocarcinoma: retrospective multicentre analysis
Source: BJS Open. 2026 Jun 8;10(3):zrag048. doi: 10.1093/bjsopen/zrag048 (PMC13243707; doi:10.1093/bjsopen/zrag048)
Supplement: zrag048_Supplementary_Data [file zrag048_supplementary_data.docx]

Preoperative biliary drainage in perihilar cholangiocarcinoma :
retrospective multicentre analysis .

Julien A. Luyten (MD)^1,2^ , Pim B. Olthof (MD, PhD)^4,5,6^, Sander M.J. van Kuijk (PhD)^7^, Silvio Nadalin (MD, PhD)^8^, Hauke Lang (MD, PhD)^9^, Ruslan Alikhanov (MD, PhD)^10^, Joris I. Erdmann (MD, PhD)^11^, Johann Pratschke (MD, PhD)^12^, Shishir K. Maithel (MD, PhD)^13^, Roberto Troisi (MD, PhD)^14^, Andreas A. Schnitzbauer (MD, PhD)^15^, Ernesto Sparrelid (MD, PhD)^16^, Peter Lodge (MD, PhD)^17^, Massimo Malagò (MD, PhD)^18^, Ulf P. Neumann (MD, PhD)^3^, Erik Schadde (MD, PhD)^19^, Hassan Z. Malik (MD, PhD)^20^, Keith J. Roberts (MD, PhD)^21^, Baki Topal (MD, PhD)^22^, Frederik J.H. Hoogwater (MD, PhD)^23^, Jeroen Hagendoorn (MD, PhD)^24^, Andrea Ruzzenente (MD, PhD)^25^, Concepcion Gomez (MD, PhD)^26^, William R. Jarnagin (MD, PhD)^27^, Matteo Cescon (MD, PhD)^28^, Luca Aldrighetti (MD, PhD)^29^, Bas Groot Koerkamp (MD, PhD)^5^, Maxime J.L. Dewulf (MD, PhD)^1#^, Steven W.M. Olde Damink (MD, PhD)^1,2,3#^, on behalf of the Perihilar Cholangiocarcinoma Collaboration Group

1. Department of Surgery, Maastricht University Medical Centre, Maastricht, The Netherlands.
2. NUTRIM, School of Nutrition and Translational Research in Metabolism, Maastricht University, Maastricht, The Netherlands.
3. Department of General, Visceral, Vascular and Transplantation Surgery, University Hospital Essen, Essen, Germany.
4. Department of Hepatobiliary, Endocrine and Transplantation Surgery, Antwerp University Hospital, Edegem, Belgium.
5. Department of Surgery, Erasmus MC Cancer Institute, Rotterdam, The Netherlands.
6. Department of Surgery, Amsterdam UMC, Amsterdam, The Netherlands.
7. Department of Clinical Epidemiology & Evaluation of Medical Technology, Maastricht University Medical Centre, Maastricht, The Netherlands.
8. Department of General and Transplant Surgery, University Hospital Tübingen, Tübingen, Germany.
9. Department of General, Visceral and Transplantation Surgery, University Medical Center Mainz, Mainz, Germany.
10. Department of Hepato-Pancreato-Biliary Surgery, Moscow Clinical Scientific Center, Moscow, Russia.
11. Department of Surgery, Cancer Center Amsterdam, Amsterdam UMC, University of Amsterdam, Amsterdam, The Netherlands.
12. Department of Surgery, Campus Charité Mitte, Campus Virchow-Klinikum, Experimental Surgery and Regenerative Medicine, Charité, Berlin, Germany.
13. Division of Surgical Oncology, Department of Surgery, Winship Cancer Institute, Emory University, Atlanta, USA.
14. Division of Hepato-Bilio-Pancreatic, Minimally Invasive and Robotic Surgery, Department of Clinical Medicine and Surgery, Federico II University Hospital, Naples, Italy.
15. Department of General and Visceral Surgery, University Hospital, Goethe University, Frankfurt, Germany.
16. Division of Surgery and Oncology, Department of Clinical Science, Intervention and Technology, Karolinska Institutet, Karolinska University Hospital, Stockholm, Sweden.
17. St James's University Hospital, Leeds Teaching Hospitals NHS Trust, Leeds, UK.
18. Department of HPB- and Liver Transplantation Surgery, University College London, Royal Free Hospitals, London, United Kingdom.
19. Department of Surgery, Cantonal Hospital Winterthur, Zurich, Switzerland.
20. Department of Hepatobiliary Surgery, Aintree University Hospital, Liverpool University Hospitals, NHS Foundation Trust, Liverpool, United Kingdom.
21. Department of Surgery, University Hospital Birmingham, Birmingham, United Kingdom.
22. Abdominal Surgery, UZ Leuven, Leuven, Belgium.
23. Department of Hepato-Pancreato-Biliary Surgery and Liver Transplantation, University Medical Center Groningen, Groningen, The Netherlands.
24. Department of Surgery, Regional Academic Cancer Centre Utrecht, St Antonius Hospital, Nieuwegein and University Medical Centre Utrecht, Utrecht, The Netherlands.
25. Department of Surgery, Unit of Hepato-Pancreato-Biliary Surgery, University of Verona Medical School, Verona, Italy.
26. Department of HPB Surgery and Transplants, Vall d'Hebron Hospital Universitari, Vall d'Hebron Institut de Recerca (VHIR), Vall d'Hebron Barcelona Hospital Campus, Universitat Autónoma de Barcelona, Barcelona, Spain.
27. Hepatopancreatobiliary Service, Department of Surgery, Memorial Sloan Kettering Cancer Center, New York, New York, USA.
28. General Surgery and Transplantation Unit, Azienda Ospedaliero-Universitaria di Bologna, Bologna, Italy.
29. Hepato-Biliary Surgery Division, Ospedale San Raffaele-IRCCS, Milan, Italy.

.

**Corresponding author:** Maxime J.L. Dewulf, Department of Surgery, Maastricht University Medical Centre, P. Debyelaan 25, 6229 HX. Maastricht, The Netherlands **ORCID: 0000-0002-1945-5481**

**Supplementary Materials - Index**

| **Supplementary Results** |  |
| --- | --- |
| Table S1: Complete propensity score weighted uni- and multivariable analysis | *page 5* |
| Table S2: Overview of imputed covariates and outcomes, missing data amounts, and the method of imputation applied.  And Additional information regarding imputation. | *page 6-7* |
| Figure S1. Baseline variables balance before  and after propensity score weighting. | *page 8* |
|  |  |
|  |  |

**Supplementary Results**

*Table S1: Complete propensity score weighted uni- and multivariable analysis*

| **Post-hepatectomy liver failure grade B+C^1*^** | **Univariable** | **Multivariate** | | |
| --- | --- | --- | --- | --- |
| **Variable** | **p value** | **Odds Ratio** | **95% CI** | **p value** |
| ASA (III-IV)^*^ | 0.004 | 1.9 | 1.27-2.84 | **0.002** |
| Preoperative biliary drainage | 0.005 | 2.13 | 1.29-3.54 | **0.003** |
| Preoperative cholangitis^*^ | 0.007 | 1.22 | 0.89-1.68 | 0.216 |
| Bilirubin at presentation (>150 µmol L or 8.8 mg dL)^*^ | 0.029 | 1.99 | 1.17-3.40 | **0.011** |
| Portal venous embolisation | 0.050 | 0.67 | 0.41-1.10 | 0.113 |
| Hepatectomy (major) | 0.109 | 1.32 | 0.86-2.02 | 0.198 |
| Hepatic artery reconstruction^*^ | 0.120 | 0.63 | 0.31-1.29 | 0.207 |
| Pancreatoduodenectomy | 0.403 | — | — | — |
| Primary sclerosing cholangitis | 0.426 | — | — | — |
| Sex (female) | 0.466 | — | — | — |
| CA19 9 levels (>100 U mL) | 0.471 | — | — | — |
| Age (>65)^*^ | 0.581 | — | — | — |
| Bismuth Corlette classification (III-IV)^*^ | 0.718 | — | — | — |
| Resection of segment I | 0.785 | — | — | — |
| BMI (>30 kg/m²) | 0.938 | — | — | — |
| Portal venous reconstruction^*^ | 0.998 | — | — | — |
|  |  |  |  |  |
| **Major postoperative complication  (Clavien-Dindo ≥ III)** | **Univariable** | **Multivariate** | | |
| **Variable** | **p value** | **Odds Ratio** | **95% CI** | **p value** |
| Preoperative cholangitis | <0.001 | 1.89 | 1.34-2.68 | **<0.001** |
| Pancreatoduodenectomy | <0.001 | 10.84 | 2.76-42.60 | **0.001** |
| Portal venous reconstruction | <0.001 | 1.70 | 1.09-2.65 | **0.019** |
| Portal venous embolisation | 0.007 | 0.53 | 0.29-0.99 | **0.048** |
| Primary sclerosing cholangitis | 0.037 | 3.04 | 1.13-8.16 | **0.028** |
| Preoperative biliary drainage | 0.040 | 1.21 | 0.85-1.73 | 0.291 |
| Hepatectomy major | 0.133 | 1.14 | 0.79-1.64 | 0.489 |
| Hepatic artery reconstruction | 0.202 | — | — | — |
| Resection of segment I | 0.304 | — | — | — |
| BMI (>30 kg/m²) | 0.319 | — | — | — |
| Bilirubin (>150 µmol L or 8.8 mg dL) | 0.400 | — | — | — |
| Age (>65) | 0.440 | — | — | — |
| Bismuth Corlette classification (III-IV) | 0.457 | — | — | — |
| CA19 9 levels (>100 U mL) | 0.498 | — | — | — |
| Sex (female) | 0.771 | — | — | — |
| ASA (III-IV) | 0.807 | — | — | — |

After univariable screening, all variables with a P value below 0.20 were entered into the multivariable model.Variables marked with an asterisk (*) were imputed. P-values in **bold** indicate statistical significance (p ≤ 0.05). ^1^ as defined by the International Study Group of Liver Surgery. ^a^ Abbreviations; BMI, Body Mass Index; ASA, American Society of Anesthesiologists Classification

*Additional information regarding imputation*

To ensure robust imputation, this process was conducted on the dataset prior to excluding any patients, thereby maximising the dataset size. In the same fashion, data was recoded after imputation to maintain consistency. For example, imputation was done on the ASA variable with its four levels and only afterwards recoded as ASA I+II and ASA III+IV. Details on the imputed variables and outcomes, the amount of missing data and the imputation methods are in displayed in Table S1.

For all weighting variables, missing data did not exceed 15%, except for bilirubin at presentation which was 30%. The maximum percentage of missing data on secondary outcomes was 2%, except for postoperative bleeding which was 13%. Further details on the imputed variables, the amount of missing data and the imputation method are provided in Table S2

While most variables had only single-digit percentages of missing data, bilirubin at presentation had approximately 30% missing data. As such, the impact of imputation was more substantial. We did not perform a sensitivity analysis of the imputation, since bilirubin was imputed as a continuous variable and then dichotomised at a threshold. Sensitivity analysis is not directly applicable once the variable is transformed in this way. Instead, we checked the proportions of imputed and observed values to ensure consistency. Bilirubin at presentation was dichotomised at 150 µmol/L (≈8.8 mg/dL). This threshold was selected a priori based on commonly used cut-offs in Western pCCA literature, including studies evaluating biliary drainage strategies, and because it provided a clinically meaningful definition of cholestasis while retaining sufficient numbers of undrained patients for PS modelling.

*Table S2: Overview of imputed covariates and outcomes, missing data amounts, and the method of imputation applied.*

| **Variables** | **Undrained Missing data %(N)** | **Drained  Missing data %(N)** | **Method of  imputation** |
| --- | --- | --- | --- |
| Age | 13.4% (47) | 10.5% (181) | Predictive mean matching |
| ASA | 8.6% (30) | 9.4% (161) | Polytomous regression |
| Bilirubin at presentation | 23.4% (82) | 31.4% (539) | Predictive mean matching |
| Cholangitis  pre-operative | 6.3% (22) | 8.3% (142) | Logistic regression |
| Bismuth-Corlette classification | 5.4% (19) | 2.1% (36) | Polytomous regression |
| PV reconstruction | 3.1% (11) | 4.9% (84) | Logistic regression |
| HA reconstruction | 3.1% (11) | 5.3% (91) | Logistic regression |
| Major postoperative complication  (Clavien-Dindo ≥ III) | 0.9% (3) | 0.2% (4) | Logistic regression |
| PHLF | 1.4% (5) | 0.2% (3) | Polytomous regression |
| Postoperative bile leak grade | 0.9% (3) | 0.1% (1) | Polytomous regression |
| Postoperative bleeding grade | 0.9% (3) | 15.3% (263) | Polytomous regression |
| Sex | 0% (0) | 0% (0) | Not imputed |
| BMI | 18.9 % (66) | 18.0% (309) | Not imputed |
| Preoperative bilirubin | 11.1% (39) | 23.0% (395) | Not imputed |
| Primary sclerosing cholangitis | 12.3% (43) | 27.2% (467) | Not imputed |
| CA19-9 at presentation or preoperative | 45.4% (159) | 44.5% (764) | Not imputed |
| T stage (7th edition) | 3.1% (11) | 2.2% (36) | Not imputed |
| N stage (7th edition) | 2.9 (10) | 2.1% (36) | Not imputed |
| M stage (7th edition) | 7.1 (25) | 9.5% (163) | Not imputed |
| Differentiation grade | 11.6% (40) | 6.7% (115) | Not imputed |
| Perineural invasion | 13.7% (48) | 12.1% (207) | Not imputed |
| POB | 0.0% (0) | 0.0% (0) | Not imputed |
| PVE | 0.0% (0) | 0.0% (0) | Not imputed |
| Type of hepatectomy | 0.0% (0) | 0.0% (0) | Not imputed |
| Segment I resected | 0.0% (0) | 0.0% (0) | Not imputed |
| Pancreatoduodenectomy | 7.1% (25) | 7.9% (135) | Not imputed |
| 90 days postoperative mortality | 0.0% (0) | 0.0% (0) | Not imputed |
| Operative blood loss | 43.4 % (152) | 49.5% (849) | Not imputed |
| Operating time | 27.1 % (95) | 26.3% (451) | Not imputed |
| Overall survival | 0.3 % (1) | 1.0% (17) | Not imputed |

Values in parentheses are absolute numbers unless indicated otherwise Abbreviations; BMI, Body Mass Index; ASA, American Society of Anesthesiologists Classification; POB, Preoperative Biliary Drainage; PVE, Portal Vein Embolization; PV, Portal Vein; HA, Hepatic Artery; PHLF, Post-Hepatectomy Liver Failure; CI, Confidence Interval.

*Figure S1. Baseline variables balance before and after propensity score weighting.*

**
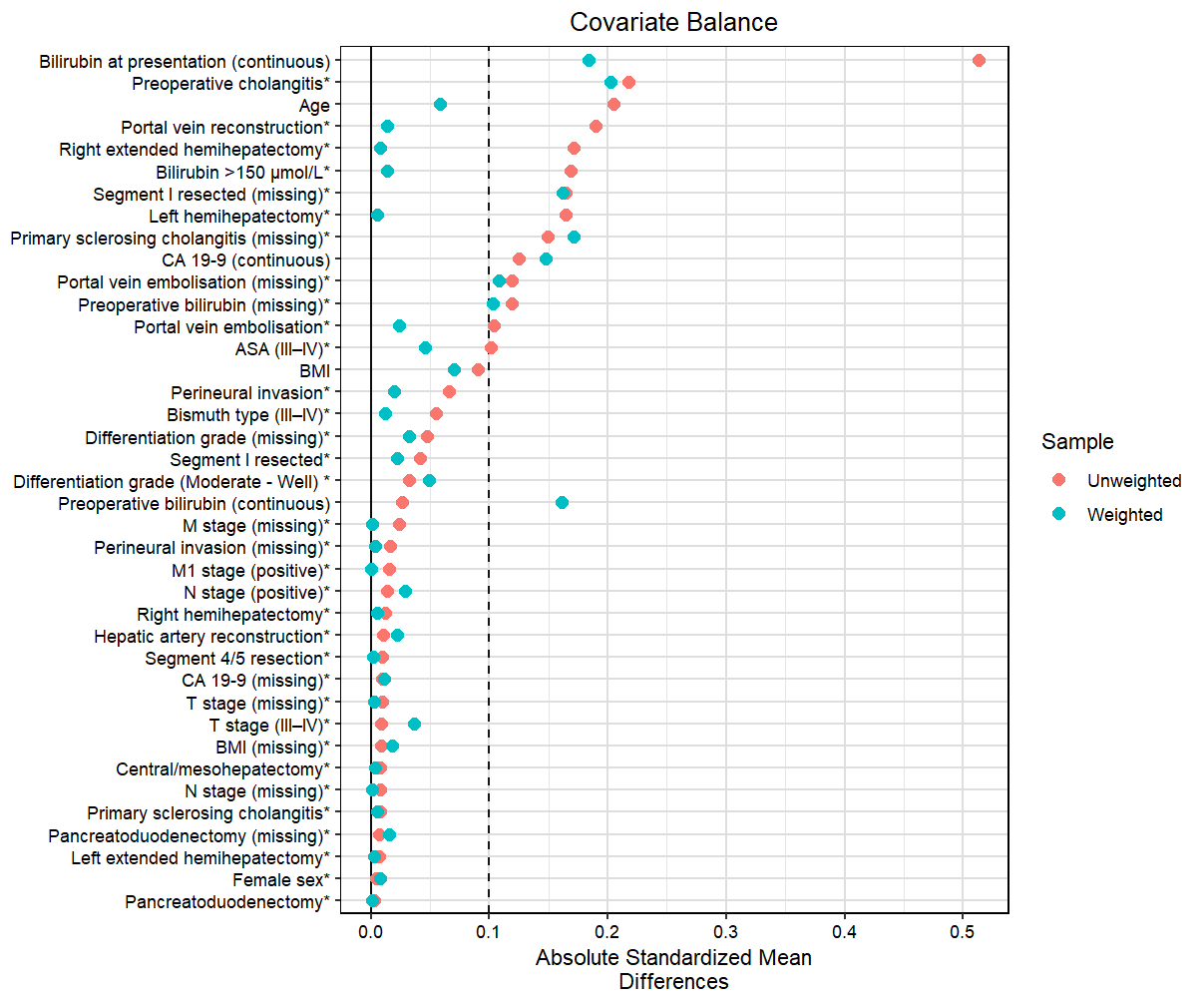
**

Absolute standardized mean differences (SMDs) for all baseline variables are shown before (red) and after (blue) propensity score weighting. For variables containing missing values, imbalance in the proportion of missing data is also displayed and labelled as “(missing)”. The dashed line indicates the conventional threshold for acceptable balance (SMD < 0.10). Abbreviations; BMI: Body mass index,
